# Opioid prescription status around surgery, bone metastasis, or death events among patients with breast cancer in Japan: an analysis of the Japanese public health insurance comprehensive claims database (the National Database)

**DOI:** 10.1093/jjco/hyae120

**Published:** 2024-08-28

**Authors:** Manami Yoshida, Mitsunori Miyashita, Toshiaki Saeki, Shinzo Hiroi, Yasuhide Morioka, Kosuke Iwasaki, Eiko Shimizu

**Affiliations:** Medical Affairs, Shionogi & Co., Ltd., Tokyo, Japan; Department of Palliative Nursing, Health Sciences, Tohoku University Graduate School of Medicine, Sendai, Japan; Department of Breast Oncology, Saitama Medical University International Medical Center, Saitama, Japan; Medical Affairs, Shionogi & Co., Ltd., Tokyo, Japan; Medical Affairs, Shionogi & Co., Ltd., Tokyo, Japan; Social Cooperation Program of IT Healthcare, Graduate School of Pharmaceutical Sciences, The University of Tokyo, Tokyo, Japan; Social Cooperation Program of IT Healthcare, Graduate School of Pharmaceutical Sciences, The University of Tokyo, Tokyo, Japan

**Keywords:** breast cancer, Japan, longitudinal study, opioid, real-world data

## Abstract

**Objective:**

To investigate the opioid prescription status around clinical events among patients with breast cancer in Japan using a comprehensive claims database.

**Methods:**

This was a retrospective cohort study using the National Database (April 2009–March 2020). The target patients had a first breast cancer diagnosis in April 2010 or later. The percentages of patients prescribed opioids before and after surgery, before and after bone metastasis, and before death with a breast cancer diagnosis in the same month were analyzed by month and by clinical facility characteristics and location.

**Results:**

We identified 1 085 388 target patients, including 216 503, 72 645, and 70 832 patients with data for the events of surgery, bone metastasis, and death, respectively. The percentage of patients prescribed opioids in the month of surgery was the highest of the entire study period at ≥70%. The percentage of patients prescribed opioids increased before bone metastasis, peaked 1 month later, and decreased thereafter while remaining higher than that before the event. The percentage of patients who were prescribed opioids before death increased over time, peaking at 33.4% 1 month before death. Prescriptions differed by facility characteristics and facility location around surgery; no differences by facility characteristics, including location, were noted around the other events. The percentage of patients prescribed opioids was consistently lower than that reported in other countries for all events.

**Conclusions:**

We showed the opioid prescription status around clinical events, including some distinct patterns depending on facility characteristics for the period around surgery, among patients with breast cancer in Japan.

## Teaser

Opioid prescription status was examined around clinical events among patients with breast cancer using a Japanese national database. Some distinct patterns, depending on facility characteristics, were found around surgery.

## Introduction

Opioids are the most common treatment for cancer pain worldwide, but their use is reported to be less frequent in Japan than in other countries [1,2]. Breast cancer is the most common cancer among women in Japan [3] and worldwide [4]. Breast cancer patients in Japan have a relatively good prognosis, with a 10-year survival rate of ~80% [5,6], which may be associated with prolonged pain control with opioids. Given the need for prolonged pain control and the difference in opioid prescription status between Japan and other countries, it is necessary to investigate the opioid prescription status of patients with breast cancer in Japan.

We previously explored opioid prescription status around surgery, bone metastasis, and death for breast cancer patients using claims data [7]. However, we could not track when patients changed facilities because the study used hospital-based data. Therefore, although breast cancer patients may change medical facilities during treatment, the previous study did not track prescription status during the entire treatment period. Additionally, the facilities in the previous study were limited to Diagnosis Procedure Combination (DPC) hospitals, which are acute hospitals that have adopted the Japanese Diagnosis Procedure Combination/Per-Diem Payment System. Considering that most acute care beds are in DPC hospitals [8], the previous study illustrated the opioid prescription status in Japan to a certain extent. However, healthcare professionals at other kinds of hospitals also prescribe opioids, and their prescription status may differ from that of professionals in DPC hospitals. Moreover, to provide high-quality cancer care, hospitals designated cancer care hospitals by the national government (hereafter referred to as designated cancer care hospitals) have been established for cancer treatment and palliative care in each prefecture and treatment area [9], totaling 445 hospitals as of 2020 [10]. The pain management performed by these hospitals may differ from that of hospitals not designated cancer care hospitals (non-designated cancer care hospitals). Facility location may also contribute to the difference in opioid prescriptions, given previous reports that the amount of some opioids differed between prefectures [2,10,11]. Although the purpose of opioid prescriptions was not specified in these studies, regional differences may exist for the same clinical events in breast cancer patients.

We investigated the opioid prescription status around clinical events—before and after surgery, before and after bone metastasis, and before death. We used a nationwide Japanese claims database, the National Database of Health Insurance Claims and Specific Health Checkups of Japan (NDB), developed by the Japanese Ministry of Health, Labour and Welfare. The database consists of data for almost all (≥95%) health insurance claims issued in Japan [12]. Therefore, the entire treatment course could be followed. Additionally, we obtained information on facility characteristics—DPC hospital status, designated cancer-care hospital status, and the number of beds—and location by prefecture and examined differences based on these categories.

## Materials and methods

### Study design, data source, and patient identification

This was a longitudinal study using the NDB (April 2009–March 2020), including medical, DPC, and pharmacy claims data from all types of public health insurance in Japan.

Breast cancer patients were defined as women who had a definitive breast cancer diagnosis (hereafter, *diagnosis* represents *definitive diagnosis*). Among these patients, the target patients of this study were identified as those who had a first breast cancer diagnosis in April 2010 or later. The database includes the first date of medical care for each disease recorded at each medical facility; the earliest first date of medical care for breast cancer was defined as the first breast cancer diagnosis for each patient. Breast cancer diagnosis was defined by standard disease names according to code C50 of the International Classification of Diseases, 10th Revision [13].

### Items for analysis

We investigated the percentage of patients who were prescribed opioids before and after surgery or bone metastasis or before death after the first breast cancer diagnosis.

For surgery, the target patients included those with a record of breast cancer-related surgery (Supplementary Table 1). Among surgeries in DPC hospitals with DPC claims that were mainly recorded during DPC hospitalization, only those that were recorded in “Medical Related Records (SK Records)”, which were used to determine a diagnostic group for bundled payments, were included in the dataset. Patients whose first bone metastasis diagnosis after surgery was recorded during the observation period were excluded because opioid prescriptions may have been affected by the metastasis. The index month was defined as the month of the earliest breast cancer-related surgery included in the dataset.

For bone metastasis, the target patients were those with a record of the first bone metastasis diagnosis after the first breast cancer diagnosis. The bone metastasis diagnosis was defined by standard disease names (Supplementary Table 1). The index month was defined as the month of the first bone metastasis diagnosis.

For death, the target patients were those with an outcome recorded as death and a breast cancer diagnosis in the month of death. The deaths included deaths due to breast cancer and other causes. The index month was defined as the month of death.

### Analysis

Descriptive statistics were used for our analysis. The percentage of patients prescribed opioids was analyzed by month from the month of each event. The percentage was calculated using the number of target patients for each event with an observation period in the month as the denominator and the number of those patients prescribed opioids as the numerator. Opioids included strong opioids (morphine, hydromorphone, oxycodone, fentanyl, tapentadol, methadone, and buprenorphine) and weak opioids (codeine, tramadol, and pentazocine), which were defined by generic names (Supplementary Table 1) according to Japanese clinical guidelines for cancer pain management [14].

Subgroup analyses were performed based on the characteristics or location of the medical facilities that comprised the record of the index month. The subgroups were (1) DPC or non-DPC hospitals, (2) designated cancer-care hospitals or non-designated cancer-care hospitals, (3) the number of beds (no beds and <99, 100–199, 200–200, 300–499, and ≥500 beds), and (4) prefecture. Patients with the index month in facilities without information on facility characteristics were excluded from subgroup analyses.

## Results

### Patients

**Figure 1:**
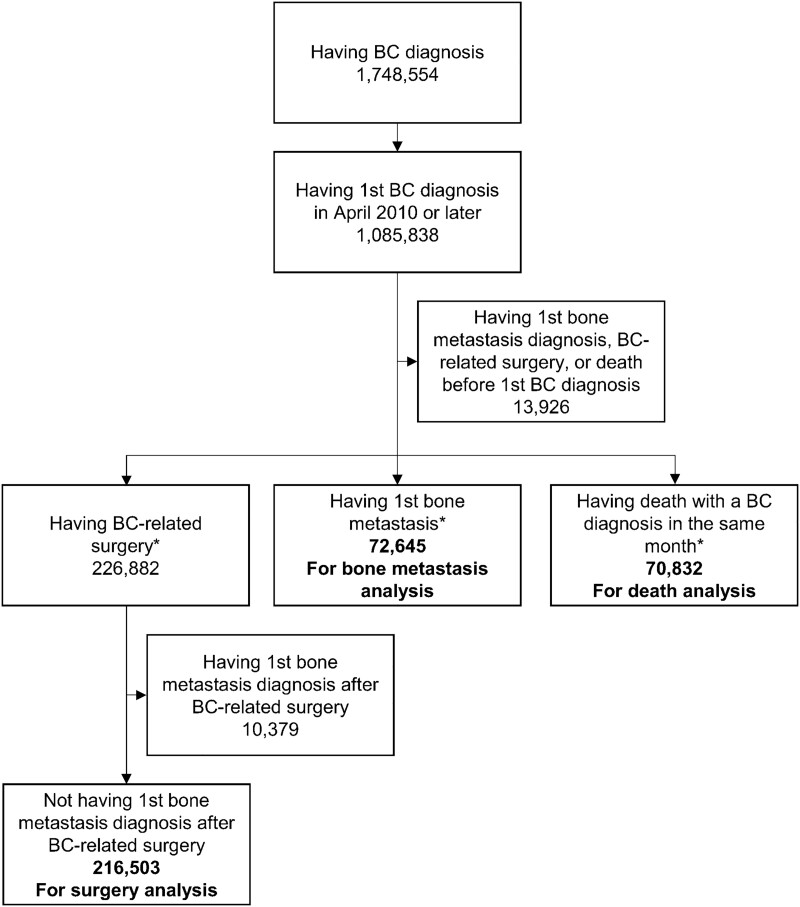
Flow diagram of the identification of target patients for analyses; the number of target patients for each event is shown in bold; diagnosis represents a *definitive diagnosis*; ^*^patients are not mutually exclusive; BC, breast cancer.

The database included 1 748 544 breast cancer patients, and 1 085 388 patients were identified as the target patients (Fig. 1). The number of target patients for each analysis and that for each subgroup are shown in Fig. 1 and Supplementary Tables 2–4, respectively.

### Opioid prescription status before and after breast cancer-related surgery

**Figure 2:**
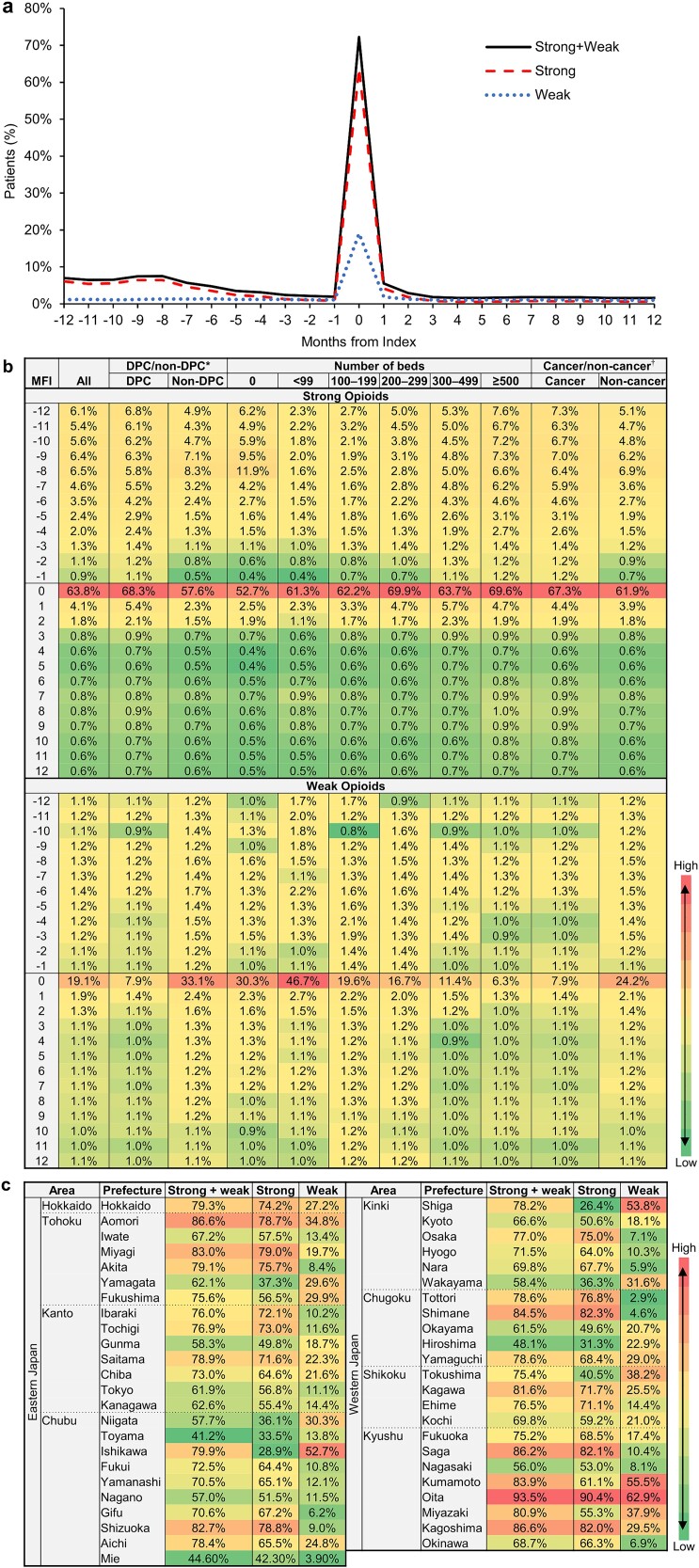
Percentage of patients prescribed opioids before and after breast cancer-related surgery by month among target patients (a) and for subgroups by medical facility (b) and prescribed opioids in the month of surgery by prefecture (c); colors are assigned for the percentage of patients prescribed opioids within each figure (Fig. 2b) or each column (Fig. 2c), from low to high, as indicated by the color bar; ^*^DPC/non-DPC represents DPC hospitals or non-DPC hospitals; †cancer/non-cancer represents designated cancer-care hospitals or non-designated cancer-care hospitals.

The number of patients targeted for surgery was 216 503 (Fig. 1). The percentage of patients prescribed strong opioids was greater than that of patients prescribed weak opioids around the surgery, with the highest percentages in the index month (Fig. 2a). The percentage in the index month was 72.3% for either strong or weak opioids and 63.8% for strong opioids (Fig. 2a and b). Before the index month, strong opioids were prescribed for 5%–6% of patients 8–12 months before, and then, the percentage tended to decrease until 1 month before; weak opioids were prescribed continuously to ~1% of patients. The percentage of patients prescribed weak opioids was 1.9% one month after the index month and ~1% thereafter; the percentages prescribed strong opioids were 4.1% one month after, 1.8% two months after, and <1% thereafter (Fig. 2b).

In terms of facility characteristics, the percentage tended to differ in the index month but did not differ in the other periods. In the index month, the percentage of patients prescribed strong opioids was ~10% higher in DPC hospitals than in non-DPC hospitals, whereas the percentage of patients prescribed weak opioids was ~25% higher in non-DPC hospitals (Fig. 2b). Facilities with 200–299 beds and those with ≥500 beds prescribed strong opioids for a larger percentage of patients, whereas those with no beds prescribed strong opioids for the smallest percentage. The percentage of patients prescribed weak opioids was ~25%–40% higher in hospitals with <99 beds, followed by those with no beds, whose percentage was ~10%–20% higher than that in other categories. In terms of location, the percentage of patients prescribed opioids tended to be higher in the areas of Tohoku and Kyushu (Fig. 2c). The difference in the highest and lowest percentages of patients prescribed opioids among the prefectures was 64% for strong opioids and 60% for weak opioids, which was greater than the difference in facility characteristics.

### Opioid prescription status before and after the first bone metastasis diagnosis

**Figure 3:**
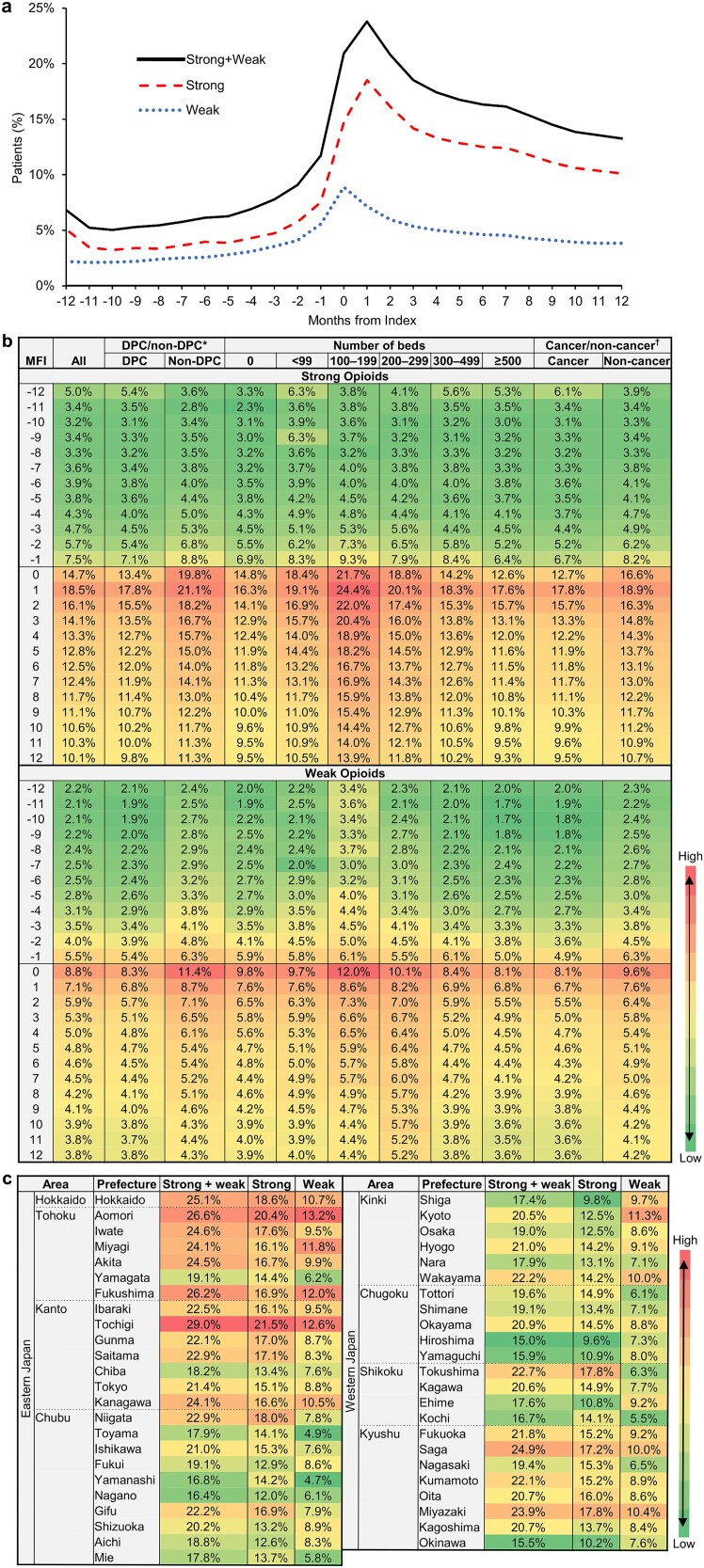
Percentage of patients prescribed opioids before and after the first bone metastasis diagnosis by month among target patients (a) and for subgroups by medical facility (b) and prescribed opioids in the month of the diagnosis by prefecture (c); colors are assigned for the percentage of patients prescribed opioids within each figure (Fig. 3b) or each column (Fig. 3c), from low to high, as indicated by the color bar; ^*^DPC/non-DPC represents DPC hospitals or non-DPC hospitals; †cancer/non-cancer represents designated cancer-care hospitals or non-designated cancer-care hospitals.

The number of patients targeted for bone metastasis was 72 645 (Fig. 1). Strong opioids were prescribed more frequently than weak opioids throughout the study period (Fig. 3a). The highest percentage of patients who were prescribed strong or weak opioids was 23.8% in the month following the index month. The percentage of patients prescribed strong opioids increased steadily to 4.7%, 5.7%, and 7.5% at 3, 2, and 1 months before the index month, respectively, and increased substantially to 14.7% in the index month, peaking at 18.5% one month after the index month (Fig. 3a and b). The percentage then decreased steadily to 10.1% at 12 months after the index month but remained higher than that before the index month. The percentage of patients prescribed weak opioids also increased steadily to 5.5% in the month prior to the index month, peaked at 8.8% in the index month, and then declined to 3.8% 12 months after the index month; however, this percentage remained higher than that before the index month.

Regarding the analysis by facility characteristics, for both strong and weak opioids, a higher percentage of patients received opioid prescriptions in non-DPC hospitals than in DPC hospitals and in non-designated cancer-care hospitals than in designated cancer-care hospitals (Fig. 3b). A higher percentage of facilities had 100–199 beds, followed by those with 200–299 beds. However, the differences were relatively small: ~5% or <4% for strong or weak opioids, respectively. The percentage was higher in eastern Japan, especially in the Hokkaido and Tohoku areas, than in western Japan (Fig. 3c). The difference in the percentage between the highest and lowest prefectures was ~10% for both strong and weak opioids.

### Opioid prescription status before death

**Figure 4:**
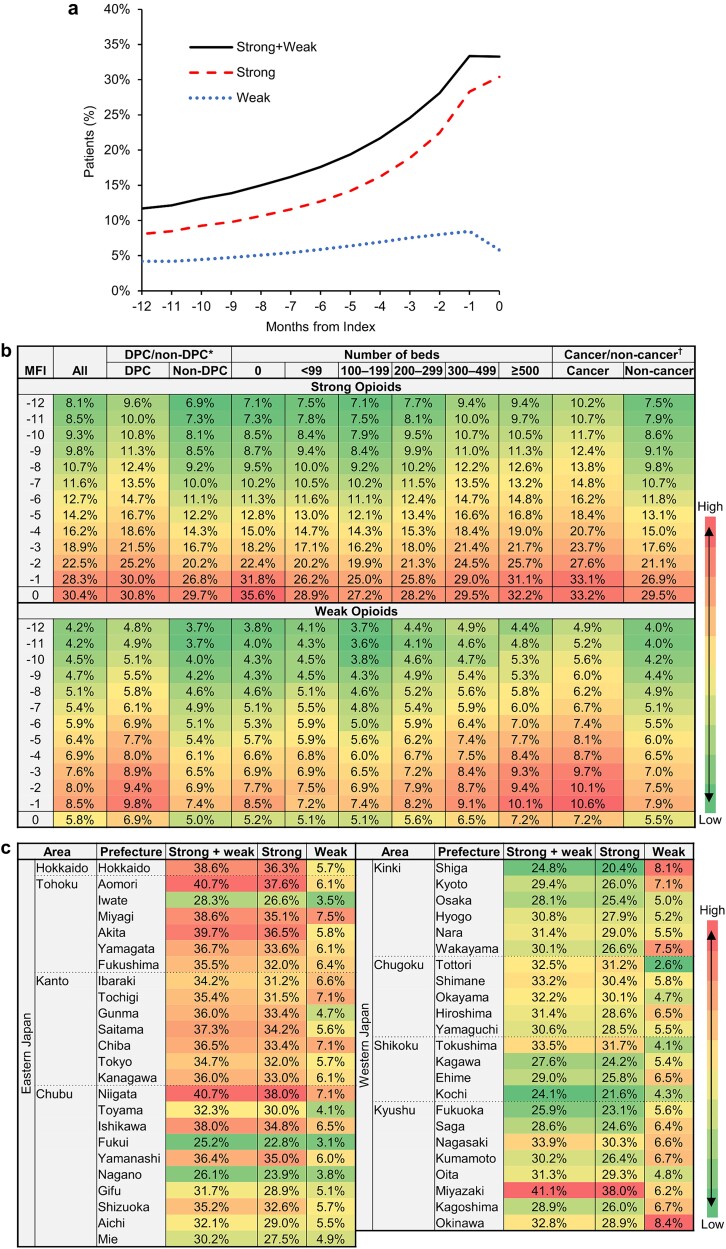
Percentage of patients prescribed opioids before death by month among target patients (a) and for subgroups by characteristics of medical facility (b) and prescribed opioids in the month of death by prefecture (c); colors are assigned for the percentage of patients prescribed opioids within each figure (Fig. 4b) or each column (Fig. 4c), from low to high, as indicated by the color bar; note: deaths include those with a breast cancer diagnosis in the same month as death; ^*^DPC/non-DPC represents DPC hospitals or non-DPC hospitals; †cancer/non-cancer represents designated cancer hospitals or non-designated cancer-care hospitals.

The number of target patients who died was 70 832 (Fig. 1). Strong opioids were prescribed more frequently than weak opioids throughout the study period; for both types of opioids, the percentage of patients increased with the month to the index month. The percentage of patients prescribed strong or weak opioids remained at 33.3%–33.4% in the month prior to and during the index month (Fig. 4a). The highest percentage for strong opioids was in the index month (Fig. 4b).

According to facility characteristics, the percentage of patients prescribed opioids was higher in DPC hospitals than in non-DPC hospitals and in designated cancer hospitals than in non-designated cancer-care hospitals for both strong and weak opioids throughout the entire study period. However, the differences were small and within ~5% for strong opioids and within ~2% for weak opioids (Fig. 4b). In terms of facility size, for strong opioids, the highest percentage was in facilities with no beds, and the second highest percentage was in facilities with ≥500 beds. For weak opioids, facilities with more beds had a higher percentage of patients prescribed opioids, although the percentage was slightly higher in facilities with no beds than in facilities with <200 beds. The percentage was also higher in eastern Japan, particularly in the Hokkaido and Tohoku areas, than in western Japan (Fig. 4c). The difference in the percentage between the highest and lowest prefectures was ~18% and ~6% for strong and weak opioids, respectively.

## Discussion

This study is the first to examine the association between opioid prescription status and clinical events in breast cancer patients using comprehensive Japanese health insurance claims data.

The percentage of patients prescribed opioids in the month of surgery was exceptionally high, with >70% of patients receiving either strong or weak opioids, including >60% receiving strong opioids. These opioid prescriptions appear to be for fentanyl for surgery [15]. However, both fentanyl used for surgery and fentanyl used for cancer pain were included, as they could not be distinguished in the data source. The percentage decreased up to 1 month prior to the index month; this decrease was also found in our previous study and was considered due to the increase in the number of target patients prior to surgery [7]. In this study, the number of patients increased from 17 620 ten months prior to surgery to 180 314 one month prior to surgery (Supplementary Table 2a). Considering that patients underwent surgery within a few months after the first breast cancer diagnosis, as indicated in this study, they may not have required opioids soon after the first diagnosis, resulting in a decrease in the percentage of prescriptions before surgery. The percentage of patients who received prescriptions for weak opioids was relatively high in facilities with no beds compared with facilities with beds. In facilities with no beds, minimally invasive ambulatory surgery may have been performed, resulting in less use of strong opioids and more frequent use of weak opioids. The percentage of prescriptions in the month of surgery varied widely among prefectures. This may be related to the fact that the distribution of anesthesiologists differs among prefectures [16]. When anesthesiologists are not available, other physicians administer anesthesia [17]. In such cases, anesthesia may differ from the standard, which may contribute to the differences in opioid prescriptions among prefectures. The percentage of patients prescribed opioids in our study was ~1% three months or longer after surgery, which was lower than that after surgery (~5%) in the USA [18]. Although the percentage of patients with persistent pain after surgery could not be examined in this study, the incidence in Japan may be different from that in other countries, which is reported to be ~50% [19,20]. The difference in this incidence may be one of the reasons for the difference in the percentage of patients prescribed opioids between Japan and the USA [18]. Notably, it is unclear whether the earliest surgery after the first diagnosis in the dataset analyzed in this study was the first surgery for a patient, as not all surgeries were recorded in the dataset.

The number of patients prescribed opioids, regardless of the type of opioid, tended to increase before the bone metastasis diagnosis, suggesting that the number of patients with pain due to bone metastasis increased closer to the bone metastasis diagnosis. However, the peak prescription percentage was observed 1 month after bone metastasis diagnosis. This is probably because soon after diagnosis, the pain may not have been severe enough to require opioids. Similar results were obtained in our previous study; the percentage of patients prescribed opioids increased before the first bone metastasis diagnosis, peaked at 1 month after the first diagnosis, and then tended to decrease over time [7]. The peak percentage was also similar between the studies: ~25% in this study and ~30% in the previous study. Although the percentage tended to decrease over time, it remained higher after bone metastasis diagnosis than before. The decreasing trend after bone metastasis diagnosis may include the possibility that pain was reduced with treatments other than opioids, such as bisphosphonates. Another possibility is that, in the long term, patients with severe pain who required opioids may have died earlier than those who did not require opioids. As reported in a previous study, the percentage of patients prescribed opioids was high in other countries; it was >50% in Canada [21], and it was >60% in the USA after any metastasis [18]. Although the percentage itself differed from that in our study, a decreasing tendency in opioid prescriptions after metastasis diagnosis was also observed, and the percentage reached ~20% in the USA [18].

In examining opioid prescription status among breast cancer patients, we analyzed deaths in which a breast cancer diagnosis was made in the same month to exclude those that occurred in patients who were in remission at the time of death. The deaths included both those due to breast cancer and those due to other causes. The percentage of patients who were prescribed opioids increased with time before death; that of patients who were prescribed either strong or weak opioids was 33.4%, and that of patients who were prescribed strong opioids was 30.4%. Regarding facility characteristics, a noteworthy difference was not observed. In our previous study, we examined opioid prescription status before breast cancer-related death using discharge summary data from DPC hospitalizations. In that study, breast cancer death was defined as a death recorded as an outcome at DPC hospitalization discharge with a record of breast cancer as any main condition, a trigger-for-hospitalization condition, or the greatest-resource-consuming condition. In that study, >70% of patients were prescribed opioids (strong or weak) in the month of death, which was much higher than in this study. This inconsistency may be because this study included a higher proportion of breast cancer patients who died from causes other than breast cancer and who were likely to be prescribed fewer opioids than patients who died due to breast cancer. Other possible reasons, such as the inclusion of inpatients at DPC hospitals, including patients with pain caused by cancer progression or metastasis in the previous study, can also be considered. In a recently published study that analyzed opioid prescriptions for patients with all types of cancer and metastasis using data from DPC hospitals, ~50% of patients were prescribed opioids prior to their death [22]. The prescription of opioids before death may vary depending on the condition of the patient with cancer. Further studies are needed to examine opioid prescription status around death among breast cancer patients considering vaqrious factors, such as the cause of death and facility characteristics, using sufficient data on deaths.

The percentages of patients prescribed opioids differed by prefecture. Some differences in palliative care status have been reported among prefectures in Japan [2,10]. The percentage of patients who died in the palliative care unit was >30% in the highest-use prefecture and 2% in the lowest-use prefecture. There were 273 palliative care physicians in Japan in 2020; one prefecture had the largest number, at 50, whereas five prefectures had zero [10]. These differences may contribute to differences in the percentage of patients prescribed opioids across prefectures. The difference in knowledge of opioid use to achieve pain relief among physicians and nurses may also be associated with these differences [2].

This study is the first to clarify the opioid prescription status among all patients with breast cancer in Japan, utilizing a national database. Differences due to facility and region, as well as comparisons with other countries, could provide useful information for future endeavors to achieve standardized and appropriate opioid usage for pain management among patients with breast cancer in Japan. However, this study has several limitations. First, because the diagnoses and medical procedures analyzed were based on database records, inaccuracies in insurance claim information may have affected the results. Second, the first diagnosis of breast cancer or bone metastasis was defined as the earliest first date of medical care recorded by each facility. The first diagnosis that occurred before the observation period was also recorded if the patients visited the facility during this period; however, the first diagnosis was not available for facilities that patients visited only before the observation period. Third, because data are aggregated on a monthly basis, a prescription within 1 month (30 days) from the index date can be included in the months before and after the index month. Fourth, deaths were identified based on the outcome records, and not all deaths were reflected in these records. Thus, if a patient died but their death was not recorded in the database, the patient was still included in the denominator for each indexed event, which may have resulted in underestimation of the percentage of patients prescribed opioids. In addition, although patients with a breast cancer diagnosis in the same month of death were included as breast cancer patients who died, we could not perform analysis by cause of death because the cause was not clear for many deaths due to a lack of information in the database. Fifth, among the surgeries performed during DPC hospitalizations, only those used to determine the diagnostic group for bundled payments were included. Therefore, it was unclear whether the earliest surgery analyzed for each patient was their first. Sixth, information on facility characteristics was obtained for the subgroup analyses, but not all facilities had this information, and facilities without it were excluded. However, we believe that exclusion may not have affected the results because the availability of the information is likely to be independent of prescription status. In addition, some information may be inaccurate, leading to subgroup misclassification. Finally, although patient characteristics, such as age, may be related to opioid prescriptions, age information could not be obtained or used in the analyses. Information on the quantity of prescriptions could also not be obtained or analyzed.

## Conclusion

In addition to our previous study that reported opioid prescription status only in DPC hospitals, this study examined the status around clinical events in breast cancer patients in Japan using a comprehensive claims database, regardless of facility type. We also examined the difference in status by facility characteristics and location. The percentage of patients who were prescribed opioids was highest in the month of surgery, was higher after bone metastasis diagnosis than before, and increased before death. The percentage of patients prescribed opioids differed by facility characteristics, especially by the prefecture in which each facility was located. The percentage of patients prescribed opioids was consistently lower than that reported in other countries for all clinical events. We believe that these results provide useful information for future opioid pain management in breast cancer patients in Japan.

## Supplementary Material

Supplementary_Table1_for_Final_hyae120

Supplementary_Table2_hyae120

Supplementary_Table3_hyae120

Supplementary_Table4_hyae120

## Data Availability

The raw data that support the findings of this study were provided by the Ministry of Health, Labour and Welfare in Japan. Because access to the raw data is strictly limited to authorized researchers and the raw and interim analysis data must be deleted after the research period, the raw and interim data will not be made available to other researchers. The analytical methods and results approved for publication by the Ministry of Health, Labour and Welfare will be made available upon reasonable request.
