# Supplementary material for: Opioid prescription status around surgery, bone metastasis, or death events among patients with breast cancer in Japan: an analysis of the Japanese public health insurance comprehensive claims database (the National Database)
Source: Jpn J Clin Oncol. 2024 Aug 28;55(1):49–58. doi: 10.1093/jjco/hyae120 (PMC11708217; doi:10.1093/jjco/hyae120)
Supplement: Supplementary_Table1_for_Final_hyae120 [file supplementary_table1_for_final_hyae120.docx]

**Supplementary Table 1.** Definitions of opioids, breast cancer-related surgery, and bone metastasis

a. Opioids

| Type | Generic name | Dosage form |
| --- | --- | --- |
| Strong | Morphine Sulfate Hydrate | Oral |
| Strong | Morphine Hydrochloride Hydrate | Oral, injection, suppository |
| Strong | Hydromorphone Hydrochloride | Oral |
| Strong | Oxycodone Hydrochloride Hydrate | Oral, injection |
| Strong | Fentanyl | Topical, |
| Strong | Fentanyl Citrate | Injection, topical |
| Strong | Tapentadol Hydrochloride | Oral |
| Strong | Methadone Hydrochloride | Oral |
| Strong | Buprenorphine Hydrochloride | Injection, suppository |
| Weak | Codeine Phosphate Hydrate | Oral |
| Weak | Tramadol Hydrochloride | Oral, injection |
| Weak | Tramadol Hydrochloride/Acetaminophen Combined Drug | Oral |
| Weak | Pentazocine | Injection |
| Weak | Pentazocine Hydrochloride | Oral |

b. Breast cancer-related surgery

| Health claim code | Procedure name |
| --- | --- |
| 150371710 | Free skin transplantation (microscopically vascularized) (Breast reconstruction) |
| 150008910 | Free skin transplantation (microscopically vascularized) |
| 150371910 | Reconstruction surgery using tissue dilation device (breast [reconstruction surgery]) |
| 150255510 | Reconstruction surgery using tissue dilation device |
| 150120910 | Abscess of breast incision |
| 150121110 | Extirpation of breast tumor (less than 5 cm in diameter) |
| 150121210 | Extirpation of breast tumor (5 cm or more in diameter) |
| 150274610 | Segmental mastectomy |
| 150121410 | Mastectomy |
| 150413710 | Mastectomy (patients with hereditary breast and ovarian cancer syndrome) |
| 150121550 | Breast cancer cryoablation |
| 150121610 | Breast malignant tumor surgery (simple mastectomy [breast removal]) |
| 150121710 | Breast malignant tumor surgery (mastectomy and breast muscle resection ae not performed simultaneously) |
| 150121810 | Breast malignant tumor surgery (mastectomy and breast muscle resection are performed simultaneously) |
| 150121910 | Breast malignant tumor surgery (extended mastectomy [dissection performed simultaneously]) |
| 150262710 | Breast malignant tumor surgery (partial mastectomy [with axillary lymph node dissection]) |
| 150303110 | Breast malignant tumor surgery (partial mastectomy [without axillary lymph node dissection]) |
| 150316510 | Breast malignant tumor surgery (mastectomy [without axillary lymph node dissection]) |
| 150386410 | Breast malignant tumor surgery (areola preservation postmastectomy [without axillary lymph node dissection]) |
| 150386510 | Breast malignant tumor surgery (areola preservation postmastectomy [with axillary lymph node dissection]) |
| 150122150 | Breast malignant tumor surgery and bilateral axillary lymph node dissection |
| 150292210 | Mammillaplasty for inverted nipples |
| 150292310 | Reconstruction mammoplasty and mammillaplasty |
| 150316610 | Breast reconstruction using artery/muscle flap (after mastectomy) (primary) |
| 150316710 | Breast reconstruction using artery/muscle flap (after mastectomy) (secondary) |
| 150369750 | Breast reconstruction (artificial breast) (primary one-stage surgery) |
| 150369850 | Breast reconstruction (artificial breast) (primary two-stage surgery or secondary) |
| 150374010 | Breast reconstruction using gel filled artificial breast (after mastectomy) |

c. Bone metastasis

| Standard disease name |
| --- |
| Bone metastasis in cancer |
| Metastatic bone tumor |
| Breast cancer bone metastasis |
