# Supplementary material for: Opioid prescription status around surgery, bone metastasis, or death events among patients with breast cancer in Japan: an analysis of the Japanese public health insurance comprehensive claims database (the National Database)
Source: Jpn J Clin Oncol. 2024 Aug 28;55(1):49–58. doi: 10.1093/jjco/hyae120 (PMC11708217; doi:10.1093/jjco/hyae120)
Supplement: Supplementary_Table2_hyae120 [file supplementary_table2_hyae120.docx]

**Supplementary Table 2.** Number of target patients for surgery by facility characteristics (a) and by prefecture (b)

a

| MFI | All | Characteristics of facilities | | | | | | | | | |
| --- | --- | --- | --- | --- | --- | --- | --- | --- | --- | --- | --- |
|  |  | DPC/non-DPC | | Number of beds | | | | | | Cancer/non-cancer | |
|  |  | DPC | Non-DPC | 0 | <99 | 100–199 | 200–299 | 300–499 | ≥500 | Cancer | Non-cancer |
| -12 | 14,203 | 8,741 | 5,024 | 3,235 | * | 984 | * | 2,244 | 5,803 | 6,128 | 7,637 |
| -11 | 15,653 | 9,635 | 5,525 | 3,606 | 687 | 1,047 | 916 | 2,459 | 6,445 | 6,806 | 8,354 |
| -10 | 17,620 | 10,833 | 6,227 | 4,115 | * | * | 1,000 | 2,750 | 7,286 | 7,707 | 9,353 |
| -9 | 20,462 | 12,743 | 7,044 | 4,573 | 847 | 1,395 | 1,176 | 3,282 | 8,514 | 9,067 | 10,720 |
| -8 | 25,474 | 16,135 | 8,350 | 5,120 | 1,157 | 1,870 | 1,568 | 4,223 | 10,547 | 11,409 | 13,076 |
| -7 | 31,667 | 19,998 | 10,258 | 5,857 | 1,730 | 2,388 | 2,096 | 5,406 | 12,779 | 14,067 | 16,189 |
| -6 | 35,135 | 22,104 | 11,394 | 6,267 | 2,044 | 2,695 | 2,410 | 6,056 | 14,026 | 15,499 | 17,999 |
| -5 | 37,815 | 23,716 | 12,329 | 6,619 | 2,270 | 2,962 | 2,670 | 6,528 | 14,996 | 16,630 | 19,415 |
| -4 | 42,053 | 26,431 | 13,648 | 7,101 | 2,623 | 3,366 | 3,013 | 7,221 | 16,755 | 18,659 | 21,420 |
| -3 | 53,531 | 34,435 | 16,593 | 8,092 | 3,553 | 4,414 | 3,758 | 9,078 | 22,133 | 24,833 | 26,195 |
| -2 | 94,897 | 59,352 | 30,836 | 12,867 | 8,095 | 9,014 | 7,007 | 16,539 | 36,666 | 41,714 | 48,474 |
| -1 | 180,314 | 100,526 | 68,423 | 25,767 | 18,957 | 22,253 | 15,315 | 32,184 | 54,473 | 62,543 | 106,406 |
| 0 | 216,456 | 115,895 | 85,370 | 32,027 | 23,162 | 28,206 | 19,073 | 38,941 | 59,856 | 69,040 | 132,225 |
| 1 | 216,390 | 115,864 | 85,336 | 32,018 | 23,152 | 28,192 | 19,066 | 38,923 | 59,849 | 69,028 | 132,172 |
| 2 | 214,636 | 115,029 | 84,424 | 31,626 | 22,927 | 27,971 | 18,894 | 38,667 | 59,368 | 68,511 | 130,942 |
| 3 | 212,898 | 114,185 | 83,541 | 31,276 | 22,702 | 27,714 | 18,709 | 38,428 | 58,897 | 68,011 | 129,715 |
| 4 | 211,055 | 113,292 | 82,603 | 30,931 | 22,428 | 27,437 | 18,531 | 38,159 | 58,409 | 67,490 | 128,405 |
| 5 | 209,163 | 112,356 | 81,655 | 30,588 | 22,170 | 27,129 | 18,353 | 37,861 | 57,910 | 66,957 | 127,054 |
| 6 | 207,221 | 111,391 | 80,687 | 30,211 | 21,905 | 26,835 | 18,164 | 37,584 | 57,379 | 66,382 | 125,696 |
| 7 | 205,219 | 110,425 | 79,660 | 29,834 | 21,618 | 26,518 | 17,961 | 37,311 | 56,843 | 65,791 | 124,294 |
| 8 | 203,365 | 109,551 | 78,697 | 29,441 | 21,370 | 26,222 | 17,778 | 37,064 | 56,373 | 65,289 | 122,959 |
| 9 | 201,477 | 108,629 | 77,743 | 29,052 | 21,116 | 25,941 | 17,588 | 36,782 | 55,893 | 64,744 | 121,628 |
| 10 | 199,620 | 107,772 | 76,755 | 28,653 | 20,864 | 25,633 | 17,419 | 36,542 | 55,416 | 64,210 | 120,317 |
| 11 | 197,758 | 106,882 | 75,800 | 28,240 | 20,630 | 25,356 | 17,228 | 36,306 | 54,922 | 63,666 | 119,016 |
| 12 | 196,011 | 106,047 | 74,896 | 27,858 | 20,392 | 25,100 | 17,058 | 36,077 | 54,458 | 63,160 | 117,783 |

b

| Prefecture | N | Prefecture | N | Prefecture | N | Prefecture | N |
| --- | --- | --- | --- | --- | --- | --- | --- |
| Hokkaido | 14,158 | Tokyo | 27,406 | Shiga | 1,918 | Kagawa | 2,446 |
| Aomori | 2,274 | Kanagawa | 11,226 | Kyoto | 7,912 | Ehime | 2,294 |
| Iwate | 2,036 | Niigata | 1,904 | Osaka | 15,514 | Kochi | 2,188 |
| Miyagi | 4,426 | Toyama | 726 | Hyogo | 6,522 | Fukuoka | 11,769 |
| Akita | 1,341 | Ishikawa | 1,046 | Nara | 961 | Saga | 1,394 |
| Yamagata | 1,012 | Fukui | 1,018 | Wakayama | 867 | Nagasaki | 986 |
| Fukushima | 2,218 | Yamanashi | 923 | Tottori | 981 | Kumamoto | 3,841 |
| Ibaraki | 5,151 | Nagano | 2,622 | Shimane | 1,351 | Oita | 3,861 |
| Tochigi | 7,070 | Gifu | 1,687 | Okayama | 3,533 | Miyazaki | 3,890 |
| nma | 3,800 | Shizuoka | 5,123 | Hiroshima | 2,362 | Kagoshima | 2,410 |
| Saitama | 8,679 | Aichi | 11,126 | Yamaguchi | 1,930 | Okinawa | 2,805 |
| Chiba | 12,038 | Mie | 3,606 | Tokushima | 2,105 |  |  |

Note: In the column with *, the number of patients is not displayed in accordance with the guidelines for using the database, in which it is not allowed to display the number of patients below certain numbers to avoid specifying the individuals and medical institutions.
