# Supplementary material for: Opioid prescription status around surgery, bone metastasis, or death events among patients with breast cancer in Japan: an analysis of the Japanese public health insurance comprehensive claims database (the National Database)
Source: Jpn J Clin Oncol. 2024 Aug 28;55(1):49–58. doi: 10.1093/jjco/hyae120 (PMC11708217; doi:10.1093/jjco/hyae120)
Supplement: Supplementary_Table3_hyae120 [file supplementary_table3_hyae120.docx]

**Supplementary Table 3.** Number of target patients for bone metastasis by facility characteristics (a) and by prefecture (b)

a

| MFI | All | Characteristics of facilities | | | | | | | | | |
| --- | --- | --- | --- | --- | --- | --- | --- | --- | --- | --- | --- |
|  |  | DPC/non-DPC | | Number of beds | | | | | | Cancer/non-cancer | |
|  |  | DPC | Non-DPC | 0 | <99 | 100–199 | 200–299 | 300–499 | ≥500 | Cancer | Non-cancer |
| -12 | 32,430 | 25,178 | 6,533 | 3,499 | 1,745 | 2,080 | 2,218 | 9,003 | 13,166 | 15,950 | 15,761 |
| -11 | 33,188 | 25,781 | 6,665 | 3,573 | 1,787 | 2,123 | 2,268 | 9,224 | 13,471 | 16,326 | 16,120 |
| -10 | 34,027 | 26,438 | 6,815 | 3,635 | 1,882 | 2,177 | 2,331 | 9,441 | 13,787 | 16,745 | 16,508 |
| -9 | 34,924 | 27,119 | 6,988 | 3,713 | 1,961 | 2,244 | 2,385 | 9,692 | 14,112 | 17,196 | 16,911 |
| -8 | 35,835 | 27,814 | 7,172 | 3,812 | 1,998 | 2,301 | 2,456 | 9,949 | 14,470 | 17,607 | 17,379 |
| -7 | 36,773 | 28,539 | 7,343 | 3,884 | 2,057 | 2,365 | 2,506 | 10,231 | 14,839 | 18,034 | 17,848 |
| -6 | 37,743 | 29,288 | 7,523 | 3,966 | 2,104 | 2,433 | 2,576 | 10,473 | 15,259 | 18,469 | 18,342 |
| -5 | 38,708 | 30,034 | 7,687 | 4,049 | 2,133 | 2,494 | 2,644 | 10,734 | 15,667 | 18,903 | 18,818 |
| -4 | 39,851 | 30,928 | 7,900 | 4,152 | 2,186 | 2,567 | 2,707 | 11,066 | 16,150 | 19,457 | 19,371 |
| -3 | 41,519 | 32,250 | 8,153 | 4,278 | 2,245 | 2,662 | 2,801 | 11,559 | 16,858 | 20,299 | 20,104 |
| -2 | 44,657 | 34,834 | 8,579 | 4,493 | 2,337 | 2,826 | 2,964 | 12,503 | 18,290 | 21,989 | 21,424 |
| -1 | 54,633 | 43,327 | 9,718 | 5,097 | 2,616 | 3,281 | 3,512 | 15,372 | 23,167 | 27,713 | 25,332 |
| 0 | 72,590 | 57,549 | 12,734 | 6,382 | 3,364 | 4,532 | 4,914 | 20,704 | 30,387 | 36,398 | 33,885 |
| 1 | 71,346 | 56,877 | 12,206 | 6,246 | 3,236 | 4,294 | 4,767 | 20,412 | 30,128 | 36,072 | 33,011 |
| 2 | 68,406 | 54,938 | 11,288 | 5,917 | 3,019 | 3,927 | 4,478 | 19,631 | 29,254 | 35,016 | 31,210 |
| 3 | 65,824 | 53,067 | 10,628 | 5,662 | 2,854 | 3,644 | 4,238 | 18,903 | 28,394 | 33,949 | 29,746 |
| 4 | 63,724 | 51,524 | 10,125 | 5,455 | 2,741 | 3,426 | 4,070 | 18,314 | 27,643 | 33,068 | 28,581 |
| 5 | 61,762 | 50,029 | 9,705 | 5,270 | 2,636 | 3,258 | 3,916 | 17,762 | 26,892 | 32,182 | 27,552 |
| 6 | 59,998 | 48,664 | 9,338 | 5,095 | 2,548 | 3,130 | 3,791 | 17,243 | 26,195 | 31,317 | 26,685 |
| 7 | 58,324 | 47,375 | 8,980 | 4,938 | 2,458 | 2,994 | 3,654 | 16,782 | 25,529 | 30,520 | 25,835 |
| 8 | 56,891 | 46,236 | 8,719 | 4,799 | 2,379 | 2,907 | 3,553 | 16,355 | 24,962 | 29,840 | 25,115 |
| 9 | 55,397 | 45,074 | 8,424 | 4,659 | 2,299 | 2,794 | 3,451 | 15,928 | 24,367 | 29,109 | 24,389 |
| 10 | 53,875 | 43,882 | 8,119 | 4,531 | 2,215 | 2,686 | 3,322 | 15,485 | 23,762 | 28,399 | 23,602 |
| 11 | 52,559 | 42,836 | 7,880 | 4,413 | 2,158 | 2,597 | 3,221 | 15,122 | 23,205 | 27,732 | 22,984 |
| 12 | 51,309 | 41,832 | 7,660 | 4,287 | 2,106 | 2,521 | 3,131 | 14,746 | 22,701 | 27,100 | 22,392 |

b

| Prefecture | N | Prefecture | N | Prefecture | N | Prefecture | N |
| --- | --- | --- | --- | --- | --- | --- | --- |
| Hokkaido | 4,025 | Tokyo | 9,021 | Shiga | 691 | Kagawa | 403 |
| Aomori | 796 | Kanagawa | 4,917 | Kyoto | 1,349 | Ehime | 732 |
| Iwate | 578 | Niigata | 1,058 | Osaka | 5,620 | Kochi | 347 |
| Miyagi | 1,234 | Toyama | 576 | Hyogo | 2,642 | Fukuoka | 3,255 |
| Akita | 485 | Ishikawa | 590 | Nara | 764 | Saga | 441 |
| Yamagata | 529 | Fukui | 303 | Wakayama | 528 | Nagasaki | 1,090 |
| Fukushima | 801 | Yamanashi | 358 | Tottori | 296 | Kumamoto | 922 |
| Ibaraki | 1,532 | Nagano | 1,192 | Shimane | 367 | Oita | 537 |
| Tochigi | 954 | Gifu | 941 | Okayama | 860 | Miyazaki | 527 |
| Gunma | 1,144 | Shizuoka | 1,964 | Hiroshima | 2,590 | Kagoshima | 898 |
| Saitama | 3,334 | Aichi | 3,765 | Yamaguchi | 1,206 | Okinawa | 1,165 |
| Chiba | 4,121 | Mie | 776 | Tokushima | 366 |  |  |
