# Supplementary material for: Opioid prescription status around surgery, bone metastasis, or death events among patients with breast cancer in Japan: an analysis of the Japanese public health insurance comprehensive claims database (the National Database)
Source: Jpn J Clin Oncol. 2024 Aug 28;55(1):49–58. doi: 10.1093/jjco/hyae120 (PMC11708217; doi:10.1093/jjco/hyae120)
Supplement: Supplementary_Table4_hyae120 [file supplementary_table4_hyae120.docx]

**Supplementary Table 4.** Number of target patients for breast cancer death by facility characteristics (a) and by prefecture (b)

a

| MFI | All | Characteristics of facilities | | | | | | | | | |
| --- | --- | --- | --- | --- | --- | --- | --- | --- | --- | --- | --- |
|  |  | DPC/non-DPC | | Number of beds | | | | | | Cancer/non-cancer | |
|  |  | DPC | Non-DPC | 0 | <99 | 100–199 | 200–299 | 300–499 | ≥500 | Cancer | Non-cancer |
| -12 | 86,017 | 43,866 | 40,044 | 12,876 | 9,609 | 15,989 | 8,937 | 19,223 | 17,276 | 20,681 | 63,229 |
| -11 | 87,932 | 44,822 | 40,913 | 13,117 | 9,837 | 16,338 | 9,128 | 19,656 | 17,659 | 21,146 | 64,589 |
| -10 | 89,844 | 45,766 | 41,791 | 13,357 | 10,060 | 16,696 | 9,341 | 20,053 | 18,050 | 21,608 | 65,949 |
| -9 | 91,844 | 46,736 | 42,737 | 13,597 | 10,311 | 17,108 | 9,544 | 20,457 | 18,456 | 22,064 | 67,409 |
| -8 | 93,836 | 47,712 | 43,663 | 13,845 | 10,545 | 17,522 | 9,748 | 20,887 | 18,828 | 22,528 | 68,847 |
| -7 | 95,881 | 48,685 | 44,628 | 14,104 | 10,789 | 17,932 | 9,962 | 21,341 | 19,185 | 22,953 | 70,360 |
| -6 | 98,006 | 49,722 | 45,612 | 14,371 | 11,052 | 18,348 | 10,181 | 21,798 | 19,584 | 23,418 | 71,916 |
| -5 | 100,299 | 50,806 | 46,698 | 14,642 | 11,357 | 18,821 | 10,421 | 22,275 | 19,988 | 23,911 | 73,593 |
| -4 | 102,786 | 51,937 | 47,910 | 14,942 | 11,667 | 19,347 | 10,681 | 22,781 | 20,429 | 24,410 | 75,437 |
| -3 | 105,507 | 53,237 | 49,221 | 15,234 | 12,002 | 19,939 | 11,025 | 23,353 | 20,905 | 24,957 | 77,501 |
| -2 | 108,856 | 54,756 | 50,859 | 15,596 | 12,434 | 20,669 | 11,417 | 24,037 | 21,462 | 25,637 | 79,978 |
| -1 | 112,893 | 56,548 | 52,885 | 16,038 | 12,959 | 21,535 | 11,911 | 24,870 | 22,120 | 26,426 | 83,007 |
| 0 | 115,797 | 57,778 | 54,409 | 16,390 | 13,356 | 22,180 | 12,252 | 25,430 | 22,579 | 26,947 | 85,240 |

b

| Prefecture | N | Prefecture | N | Prefecture | N | Prefecture | N |
| --- | --- | --- | --- | --- | --- | --- | --- |
| Hokkaido | 5,953 | Tokyo | 12,465 | Shiga | 1,022 | Kagawa | 828 |
| Aomori | 1,466 | Kanagawa | 8,158 | Kyoto | 2,296 | Ehime | 1,477 |
| Iwate | 1,233 | Niigata | 2,265 | Osaka | 8,277 | Kochi | 733 |
| Miyagi | 2,172 | Toyama | 982 | Hyogo | 4,788 | Fukuoka | 5,329 |
| Akita | 1,104 | Ishikawa | 1,095 | Nara | 1,224 | Saga | 767 |
| Yamagata | 1,090 | Fukui | 617 | Wakayama | 900 | Nagasaki | 1,277 |
| Fukushima | 1,658 | Yamanashi | 744 | Tottori | 501 | Kumamoto | 1,590 |
| Ibaraki | 2,527 | Nagano | 1,978 | Shimane | 645 | Oita | 1,019 |
| Tochigi | 1,736 | Gifu | 1,755 | Okayama | 1,635 | Miyazaki | 931 |
| Gunma | 1,767 | Shizuoka | 3,203 | Hiroshima | 2,585 | Kagoshima | 1,477 |
| Saitama | 6,170 | Aichi | 6,022 | Yamaguchi | 1,446 | Okinawa | 972 |
| Chiba | 5,688 | Mie | 1,517 | Tokushima | 713 |  |  |
